# Immunomodulatory and Anticancer Activities of Barley Bran Grown in Jordan: An *in vitro* and *in vivo* Study

**DOI:** 10.3389/fnut.2022.838373

**Published:** 2022-05-18

**Authors:** Sara Feras Abuarab, Wamidh H. Talib

**Affiliations:** Department of Clinical Pharmacy and Therapeutic, Applied Science Private University, Amman, Jordan

**Keywords:** anticancer nutrition, immunomodulatory effect, natural products, tumors, animal model

## Abstract

The Mediterranean diet is regarded as one of the most healthful dietary patterns in the world, owing to a combination of foods high in antioxidants and anticancer constituents. Barley bran is one of the components of the Mediterranean diet. It has nutritional and beneficial effects in different pathological conditions. Many studies were achieved to assess the nutritious values of barley bran, but there is no research indicating immunomodulatory and anticancer activities of barley bran grown in Jordan. The present study aims to examine and assess the potential immunomodulatory and anti-tumor activities of ethanol, *n*-hexane, aqueous/methanol, and water extracts obtained from barley bran. The Maceration method was utilized to prepare ethanol, *n*-hexane, aqueous/methanol, and water extracts. Various phytochemical groups were determined by using qualitative phytochemical tests. The antiproliferative activity of extracts was determined against MCF-7, HCT-116, A549, and EMT6/p by the MTT assay. The Folin-Ciocalteu reagent was used to detect the total phenolic content in extracts. Furthermore, immunomodulatory activity was assessed by determining the effect of extracts on splenocytes proliferation in the presence and absence of mitogens. The nitro blue tetrazolium assay and the neutral red method were used to assess the effect of each extract on the phagocytic activity of macrophages and pinocytosis, respectively. For the *in vivo* part, three different concentrations (10, 20, and 30% w/v) of barley bran were used to test the prophylactic effect in four Balb/C mice groups inoculated with EMT6/p cell-line subcutaneously. Also, serum samples were collected to assess the effect on cytokines (IFN-gamma, IL-2, IL-4, and IL-10). Barley bran extracts inhibited cancer cell proliferation. According to immunoassays, *n-*hexane and aqueous/methanol extracts could significantly rise lymphocyte proliferation and pinocytosis activity of macrophages. The activity of phagocytosis was increased by *n-*hexane and ethanol extracts. For the *in vivo* part, the average tumor size and weight of mice given the 30% barley bran group was significantly reduced (*p* < 0.05) compared with the control group. During our study, higher levels of TH1 cytokines (IFN- γ, IL-2) and lower levels of TH2 cytokine (IL-4) and T regulatory cytokine (IL-10) were obtained due to consumption of barley bran in food. Barley bran can be used as a prophylactic agent because it has anti-cancer and immunomodulatory activities.

## Introduction

Cancer is the second cause of death in Jordan after cardiovascular diseases (1). It is a broad term disease that causes cells to divide uncontrollably. The mortality and morbidity of cancer disease are estimated to increase as the young population ages with longer life expectancy (1). Cancer can be caused by external and internal factors. External factors include radiations, smoking tobacco, and pollutants in drinking water, food, air, chemicals, certain metals, and infectious agents. On the other hand, the internal factors are genetic mutations, body immune system, and hormonal disorders (2). There are many types of cancer; colorectal cancer is stated to be the most prevalent type in males followed by breast cancer in females (3). According to the latest published annual report by Jordan's Cancer Registry (JCR) for the period 2006 to 2015, there is a rise in the number of cancer cases. Table 1 demonstrates the most common cancers in Jordan among both genders with breast cancer being the most widespread, one among females with 1,145 registered cases. Cancer is a big problem; so many pieces of research are made about it.

**Table 1:** Ten most common cancers among Jordanians, both genders, 2015^*^.

**Rank**	**Site**	**Frequency**	**Percentage (%)**
1	Breast	1,145	20.6
2	Colorectal	668	12.0
3	Lymphoma	390	7.0
4	Lung	378	6.8
5	Urinary Bladder	296	5.3
6	Thyroid	228	4.1
7	Prostate	215	3.9
8	Leukemia	200	3.6
9	Stomach	157	2.8
10	Uterus	156	2.8

* *Data from the Jordan's Cancer Registry, Ministry of Health*.

Cancerous cells can form tumors, damage the immune system, and cause other changes that avoid body function. Cancerous cells may present in one area, and then spread via the lymphatic system to other areas (4). Cancer is not a new disease. Many people suffer from cancer throughout the world. The term cancer came from a Greek word karkinos used by Hippocrates (460–370 B.C.) to identify carcinoma tumors, but he was not the first to discover this disease (5).

Cancer was first identified by the ancient Egyptians in 1,500 BC; the first form identified was breast cancer and, subsequently, other mummies with bone cancer (around 1,600 BC); in that time, treatment was only palliative, but no effective cure was found to treat it (5).

Treatments are continuously developing. Examples of methods include chemotherapy, surgery, radiation therapy, stem cell transplantation, immunotherapy, cancer vaccination, and photodynamic therapy. Treatment methods rely on the cancer stage, type, and location (6). The drugs that are used to treat cancer are unsafe, meaning that they can cause vomiting, nausea, diarrhea, fatigue, hair loss (associated with chemotherapy), oral mucositis, oral infection (candida colonization mostly), xerostomia (loss of saliva production), and osteoradionecrosis, usually related to radiation treatment of head and neck cancers, while some rectal, bladder, and bowel toxicities were detected during radiation therapy of rectal and prostate cancers (6). Also, patients with cancer can suffer from anemia, infertility, immunosuppression [defected white blood cells (WBC) numbers] and lung dysfunction (7). Continuous efforts have been made to reduce the harmful side effects of drugs that are used to treat cancer. These side effects inspired scientists to pursue new potential natural products, either on their own or as adjuvant drugs, for anti-cancer treatment with least side effects. Plant-derived products are used because they are relatively safe, simple, low cost, and less-toxic products (8).

The first serious trials were performed in the 1950s, with the discovery of anticancer agents (vinblastine, vincristine, podophyllotoxins, and vinca alkaloids), which prompted the United States National Cancer Institution (NCI) to pay more attention and establish a programmed plant collection in 1960 (9). Many of these natural species are used in our daily diet and have shown an anti-cancer and immunomodulatory effect, such as green tea (Camellia sinensis)—inhibition of metastasis, milk thistle (Silybum marianum)—caused cell-cycle arrest and 90% reduction of tumor, garlic (Allium sativum)—deceased the rate of tumor growth, and Dandelion (Taraxacum officinale)—stimulated the immune system by rising tumor necrosis factor (TNF)-α and interleukin (IL)-1α (10).

These natural products have immunomodulatory properties, leading to immunotherapy. Immunotherapy helps the immune system to recognize and kill cancer cells. It is defined as a therapy that can activate or suppress the immune system. It aims to enhance the immunological anti-cancer response by combining vaccines with immunostimulatory cytokines or by blocking the pathways used by cancer cells to suppress the response (11). Furthermore, The Mediterranean diet combines foods common in Mediterranean-bordering countries. It includes fruits, vegetables, unrefined grains, and olive oil. It has been associated with a lower incidence of colon, prostate, stomach, and breast cancer (12).

Barley (Hordeum vulgare) is a main cereal grain cultivated in temperate climates worldwide. It is found at different places in the Fertile Crescent. It is utilized as animal feed and as a part of several health foods. Barley is similar to the Latin word farina “flour,” while bran means “barley-house” (13). Barley includes a variety of phytochemicals in various amounts, which are usually determined by genotypic or environmental variables, or by the combination of both factors. Phytochemicals in barley are classified into several key classes, including phenolic acids, flavonoids, lignans, tocols, phytosterols, and folates (14). Studies showed that barley has a number of biological activities, including angiotensin-converting enzyme inhibitory activity, tyrosinase inhibitory activity, antioxidant ability, and xanthin oxidase inhibitory activity (13). Barley also has anticancer activities against different cancer cell lines such as A549 (15). A study showed that barley has immunomodulatory activity as it has bioactive components (16).

Bran is usually obtained by millers by separating the bran (the outer layer of the grain) and the embryo from the endosperm so that bran was thrown away in the milling process, leaving defatted grain to be used (17). Studies showed that the barley bran can be used before cancer development as a prophylactic agent and can also be used during cancer formation (17). Unlike chemotherapeutic agents that suppress the immune system, making the patient more susceptible to secondary diseases, barley bran can amplify the immune system (immune system boosters) (18). Phytochemicals in barley bran have not been previously tested as a combination. In this study, we hypothesized that the active phytochemicals of barley bran may have the potential to inhibit cancer cells and activate the immune system.

## Materials and Methods

### Barley Bran Supply and Extracts Preparation

Barley bran was generously donated by Engineer Amer Abu Namous Establishment for marketing natural products in Amman, and, as declared by the workers there, barley is given to them in huge packages to grind and distribute it to the shops. It was cleaned well from Weevils by spreading it under the warm sun for 5 days in a row with alternative flipping over; after that, it was retained in dry jars until use.

Different extracts were prepared by using solvents of different polarities. Ethanol (70%), n-Hexane, aqueous/methanol (70:30), and water were utilized to macerate barley bran (1L per 100 g) for 14 days at room temperature with daily stirring. After that, residue was eliminated; the supernatant was filtered and concentrated using a rotary evaporator, entirely dried using lyophilizer, and saved at −20°C until utilized.

### Qualitative Phytochemical Screening of Barley Bran Extracts

Phytochemical screening was achieved for ethanol, n-hexane, aqueous/methanol, and water extracts. Qualitative chemical screening tests were done to identify the existence of the main classes of substances, such as saponins, tannins, terpenoids, alkaloids, flavonoids, phenols, steroids, and carbohydrates. Identification of the extracted components was performed using the standard methods described (19).

### Quantitative Analysis of Barley Bran n-Hexane and Aqueous/Methanol Extracts by Liquid Chromatography-Mass Spectrometry (LC-MS)

The samples were prepared by dissolving them in 2 ml of DMSO (dimethyl sulfoxide) and finishing the 50-ml amount with acetonitrile. Each sample was centrifuged at 4,000 rpm for 2 min, and then 1 ml was moved to the autosampler. The amount of the injection was 3 μL. The study was carried out using the impact II ESI-Q-TOF system fitted with the BurkerDalotonik Elute UPLC system from BurkerDaltonik (Berman, Germany). The device worked using the Ion Source Apollo II ion funnel electrospray source (capillary voltage, 2,500 v; nebulizer gas, 2 bars; dry gas flow, 8 L/min; dry temperature, 200°C; mass accuracy, <1 ppm; mass resolution, 50,000 FRS; the TOF repetition rate, 20 kHz). A Burker's solo 2-C-18 UHPLC column (100 mm x 2.1 mm x 2 μm) was used to conduct chromatographic separation at a flow rate of 0.51 ml/min and a column temperature of 40°C. For the detection of ms/z and retention time, all standards were used.

### Cell Lines and Cell-Culturing Condition

To investigate the potential anticancer effect of barley bran extracts, five cell lines were utilized (MCF-7, HCT-116, A549, EMT6/P, and VERO). MCF-7 [(ATCC® HTB-22™) with 14 passages] is the human epithelial breast cancer cell line. HCT 116 [(ATCC® CCL-247™) with 4 passages] is the human colon carcinoma cell line. A549 [(ATCC® CCL-185™) with 9 passages] is the adenocarcinomic human alveolar basal epithelial cell line, while EMT6/p [ATCC® CRL-2755™) with 4 passages] is the mouse epithelial breast cancer cell line. VERO is the normal kidney epithelial cells extracted from an African green monkey. Doxorubicin was used as positive control. The cells were cultured in a complete medium and incubated at 37°C in 5% CO_2_, a 95% humidity incubator. The MCF-7 cell line was cultured in a complete RPMI 1640 medium, while the EMT6/P cell line was cultured in a complete MEM medium. The rest of the cell lines were cultured in complete DMEM-medium high glucose.

### Experimental Animals

This study was carried out according to standard ethical guidelines, and all of the experimental protocols got the approval by the Research and Ethical Committee at the Faculty of Pharmacy-Applied Science Private University. To accomplish this study, Balb/C mice within age range of 4–6 weeks and body weight of 23–25 grams were utilized. Wooden shaving cages were utilized as bedding to save the mice. The environmental parameters in the animal room were 25°C temperature, 50–60% humidity with continuous air ventilation.

### Antiproliferative Assay

After culturing, trypsinization, and cell counting for a particular cell line, the cells were seeded into a 96-well tissue culture plate (100 μL/well) at an exact concentration of 15,000 cells/well using a multichannel pipette. After 24 h of incubation, the media in each well was totally removed, and the attached cells were treated in triplicate with decreasing concentrations of different extracts of barley bran (5–0.078 mg/ml), resulting in a total volume of 200 μL/well. Following incubation for 48 h, An MTT [3-(4,5-Dimethylthiazol-2-yl)-2,5-diphenyltetrazolium bromide] assay kit (Bioworld, UK) was utilized to measure cell viability. The principle of the assay is to identify the reduction of MTT by mitochondrial dehydrogenase to form blue formazan crystals, reflecting normal function of the mitochondria and cell viability. The MTT assay was done by removing old media from each well, washing it with PBS, then adding 100 μl of culture media and 10 μl of thiazolyl blue tetrazolium solution. After 3 h of plate incubation, 100 μl of DMSO was added to dissolve the formazan particles that were formed in live cells. Then, the plate was incubated for 1 h, and placed on ELISA microplate absorbance reader at 550 nm to measure the optical density (OD). To calculate the percentage of survival cells and IC50 values, Microsoft Excel software was utilized.


$$\begin{array}{l} Percentage\;of\;cell\;viability\;\left(\%\right)=\\ \left(OD\;of\;treated\;cell/OD\;of\;control\;cell\right)*100 \end{array}$$

### Preparation of the Positive Control Doxorubicin

Doxorubicin (Dox) was utilized as a positive control because it has a broad spectrum of antitumor activity. It belongs to anthracyclines, which are cytotoxic agents. To prepare desired concentrations of 200 μM, the stock solutions were diluted before use by DMEM.

### Calculation of Inhibitory Concentration (IC_50_)

Half maximal inhibitory concentration (IC_50_) is the molar concentration of a substance at which there is 50% cell death in comparison to cells of negative control. Regarding the NCA plant screening programs, if the IC_50_ value of a crude extract incubated (48–72 h) is <20 μg/ml in carcinoma cells, usually, a crude extract is considered to have *in vitro* cytotoxic activity (20). The calculation and analysis of IC_50_ values were conducted by non-linear regression test in a statistical package for the social sciences (SPSS) version 22 (Chicago, Illinois).

### Total Phenolic Content (TPC) by Folin-Ciocalteu (F-C) Reagent

The amount of TPC in barley bran extracts was detected using the F-C procedure described by Roslan et al. (21). The principle of this method signifies the reaction between F-C reagent that is made of a mixture of sodium tungstate (Na_2_WO4) and sodium molybdate (Na_2_MoO4) with phenolic compounds to give a blue color. Also, F-C was found to be reactive against other antioxidants (besides phenolics) as aldehydes, ketones, thiols, unsaturated fatty acids, proteins, amino acids, amines, nucleotides, carbohydrates, vitamins, and some nitrogenous compounds (21).

Firstly, 5 mg/ml of each extract was prepared as a stock solution. Five various dilutions were also prepared from each stock as 2.5, 1.25,0.625,0.312,0.156 mg/ml. Briefly, 12.5 μL of each dilution was mixed with 250 μL of 2% sodium carbonate solution in a 96-well microplate in triplicate. They were permitted to react at RT for 5 min. Then, 12.5 μL of 50% F-C reagent was added and permitted again to stand at RT for 30 min. A plate reader was used to read the absorbance of reaction mixture at 630 nm (21). Gallic acid standard solution was used at different concentrations, ranging from.1 to 1. mg/ml in distilled water (Appendix 3.1) to produce a standard curve. Data are expressed as equivalent of gallic acid (mg) for each milliliter of each extract (21).

### Preparation of Murine Splenocytes

The spleen was removed aseptically after a Balb/C mouse was sacrificed. Spleen cells were passed through the mesh of a tissue grinder suspended in RPMI-1640 media. The cells suspension was centrifuged at 1,500 RPM and 4°C for 10 min. After centrifugation, supernatant was eliminated and cells were re-suspended with RBC cell lysis to remove red blood cells. Pipetting of the suspension was essential for many times. After 10 min of another centrifugation, the resulted pellets were re-suspended in 5-ml RPMI-1640 media, and splenocytes were available to be counted and seeded for various assays.

### Lymphocytes Proliferation Assay

#### Lymphocytes Proliferation Assay in the Presence of Con A or LPS

Based on the manufacturer's instructions, this assay was conducted using the MTT [3-(4, 5-Dimethylthiazol-2-yl)-2, 5- diphenyltetrazolium bromide] assay kit (Bioworld, UK). Briefly, splenocytes suspension was prepared in RPMI-1640 (supplemented with 50 U/ml streptomycin, 50-U/ml penicillin, and 10% FBS) and seeded into a 96-well culture plate at a specific concentration of a 2-x-106 cell/ml in the presence of 5-μg/ml Con A or 4-μg/ml LPS. Then, 100 μL of various concentrations (0.625–5 mg/ml) of barley bran extracts were added in an in-triplicate manner. The plate was incubated for 48 h under 5% CO_2_ and humidified atmosphere of 95% air at 37°C temperature. After the incubation, each well was treated with 10 μL of MTT (5 mg/ml) solution and incubated again for 4 h. After that, it was treated with 100 μL of DMSO to dissolve the developed formazan particles, and the absorbance was measured using ELISA microplate absorbance reader at 550 nm. Results were expressed as an stimulation index compared with negative control (22).

#### Lymphocytes Proliferation Assay Without the Presence of Con A and LPS

In this assay, the same procedures were performed as the previous assay but with omission of Con A and LPS addition.

### Macrophage Isolation From Peritoneal Fluid

Owing to the high presence of tissue macrophages, the peritoneal cavity is a favored site for the collection of these cells. Forty-eight h before peritoneal macrophages (PEM) collection, mice were injected intra-peritoneally with 5 ml of a 3% (w/v) brewer thioglycollate medium (23). After that, ice-cold sterile phosphate –buffer saline (pH 7.4) was utilized to isolate peritoneal macrophages (PEM). The mice were euthanized by cervical dislocation, and their abdominal cavities were visualized to introduce 5 ml of ice-cold PBS into the cavity. Following gentle massaging, the fluid was removed and put in a centrifuge tube held on ice. The process was repeated many times. After centrifugation at 1,500 RPM, 4°C for 10 min, the cell pellets were re-suspended in a completed RPMI 1640 medium.

### *In vitro* Phagocytic Assay [Nitro Blue Tetrazolium (NBT) Reduction Test]

The NBT reduction assay was assessed, depending on the method defined by Madakkannu and Ravichandra (24). In Brief, peritoneal macrophages were seeded into a 96-well tissue culture plate at a definite concentration of 5-x-10^6^ cells/well, and cultured with various concentrations of barley bran extracts (5–0.625 mg/ml) for 48 h at 37°C. After that, each well was treated with 20-μL yeast suspension (5- × -10^7^ cells/ml in PBS) and 20-μL nitro blue tetrazolium (NBT) (1.5 mg/ml in PBS), except the control wells, which were only treated with 20-μL yeast suspension (5- × -10^7^ cells/ml in PBS). Following cells were incubated for 60 min at 37°C, the supernatant was eliminated and the adherent macrophages rinsed with RPMI 1640. The cells were air-dried before 140-μL DMSO, and 120 μL of 2M KOH was added to each well. At the end, the plate was put on a microplate reader at 550 nm to measure the optical density (OD). The percentage of NBT reduction, which represented the phagocytic activity, was calculated according to the following equation:


$$\begin{array}{l} Phagocytic\;index=\left(OD\;sample-OD\;control\right)/OD\;control*100. \end{array}$$

### Pinocytic Activity Assay by the Neutral Red Method

The neutral red method was used to evaluate macrophages pinocytic activity of barley bran extracts, which were defined by Madakkannu and Ravichandran (24). Peritoneal macrophages were seeded into a 96-well tissue culture plate at an exact concentration of 5-x-10^6^ cells/well, treated with various concentrations of barley bran extracts (5–0.625 mg/ml), and then incubated at 37°C for 48 h. Thereafter, each well was treated with 100 μL of neutral red solution (7.5 mg/ml in PBS) and incubated for 2 h. Following incubation, the supernatant was eliminated, and each well was rinsed with PBS two times to get rid of neutral red that was not pinocytized by macrophage. Then, 100 μl of cell lysis solution (ethanol and.01% acetic acid at the ratio of 1:1) was added to each well to break the cells. The plate was kept at RT overnight. At the end, the microplate reader was utilized to measure the optical density (OD) at 540 nm. Pinocytic activity was described by the absolute OD values, which reveal the dye uptake (24).

### Preparation of Food for the *in vivo* Part (Experimental Diet)

**Figure 1:**
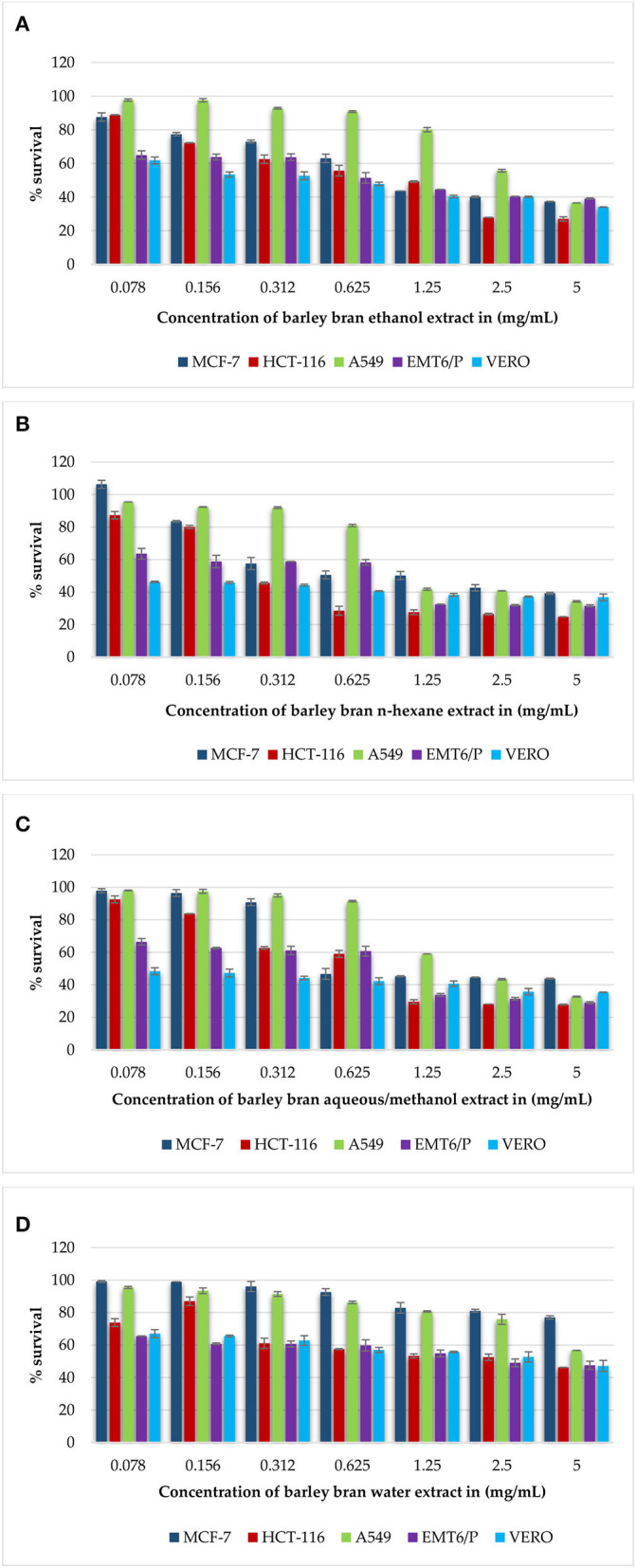
**(A–D)** Antiproliferative activity of all extracts of barley bran on cancer cell lines (MCF-7, HCT-116, A549, EMT6/P) and on the VERO normal cell line using concentrations between 0.078 and 5 mg/ml. Percentage of cell viability (%) was calculated as (OD of treated cells/OD of control cells * 100). Results are expressed as means of three independent experiments ± SD.

Three percentages were prepared by weight; the 1st group was 900-g mouse fodder and 100-gm barley bran; the 2nd group was 800-g mouse fodder and 200-g barley bran; and the 3rd group was 700-g fodder and 300-g barley bran (Figure 1).

Firstly, the fodder and barley bran were separately weighed and grinded and then well-mixed regarding to the upper categories, and then water was added to each category (the water percent was depending on the appropriate texture for kneading). After that, the dough was rolled into rollers, clipped into small similar discs, and exposed to the sun for a range of 3–5 days to dry. At the end, it was kept in a −20°C freezer until the time of usage.

### Tumor Prophylaxis Assay

Forty female Balb/C mice were divided into 4 groups: Group 1: the 10% group (were fed fodder mixed with 10% barley bran), Group 2: the 20% group (were fed fodder mixed with 20% barley bran), Group 3: the 30% group (were fed fodder mixed with 30% barley bran), and Group 4: the control group (the healthy mice were fed usual fodder and inoculated with tumor) (Table 2). This study was divided into prophylactic and treatment phases. Each phase lasted 15 days.

**Table 2:** Study groups with percentages received, an *in vivo* experiment.

**Group**	**Number of mice**	**Name**	**Prophylaxis/treatment**
Group 1	10	10% BB	10% BB in fodder
Group 2	10	20% BB	20% BB in fodder
Group 3	10	30% BB	30% BB in fodder
Group 4	10	Control group	0% BB in fodder

The feeding process began from September 29, 2020 to November 1 (the day of the sacrifice).

About 1,000,000 EMT6/p cells of tumorigenic dose per 1 ml of MEM media were injected subcutaneously (S.C.) on October 13 (2 weeks of feeding pre-inoculation) in the abdominal area of each 11–12-week-old female Balb/C mouse.

Tumors were permitted to be grown for 14 days; here, we showed the number of tumors, the size, and the weight averages of each barley bran group. After that, we compared them with the control group. Volumes were measured using the following below equation (25); digital caliper was utilized to evaluate tumors size.

Tumor volume = A^*^ B^2^^*^0.5

where:

A = length of the longest aspect of the tumor

B = length of the tumor aspect perpendicular to A.

### Determination of IFN-γ, IL-2, IL-4, and IL-10 Levels in a Serum Sample

Serum levels of IFN-γ, IL-2, IL-4, and IL-10 were evaluated for demonstrative samples of mice from all research groups using a mouse TH1/TH2 ELISA kit (affymetrixebioscience, Canada). The same quantitative sandwich enzyme immunoassay technique was applied for IFN-γ, IL-2, IL-4, and IL-10. A 96-well plate (Corning™ Costar™ 9018) was coated with a monoclonal capture antibody specific for mice IFN-γ, IL-2, IL-4, and IL-10 and incubated overnight at 4°C; after that, aspirated, washed three times, and soaked with a wash buffer for 1 min. ELISA/ELISPOT diluent was utilized to block the wells and incubated at room temperature for 1 h. Standards control and samples were pipetted into the wells and incubated at room temperature for 2 h. After washing was applied, detection antibody specific for mice IFN-γ, IL-2, IL-4, and IL-10 was added and incubated at room temperature for 1 h. After another washing, an avidin-HRP (enzyme) was added and incubated at room temperature for half an hour. After that, another washing was applied; a substrate solution of TMB (Tetramethylbenzidine) was added and incubated at room temperature for 15 min. Finally, the stop solution was added, and ELISA microplate absorbance reader was utilized to measure the absorbance at 450 nm. The intensity of the color measured is in proportion to the quantity of mouse IFN-gamma or mice IL-2, IL-4, and IL-10 bound in the initial stage. The samples absorbance values were then read off the standard curve (Appendices 3.2–3.5).

A detailed and step-by-step procedure was done according to Catalog No. 887314 for mice IFN-γ, IL-2, IL-4, and IL-10, respectively.

### Statistical Analysis

Data were expressed using the mean ± standard deviation of triplicate independent experiments utilizing the SPSS 22 one-way ANOVA, followed by post hoc test. When the *P*-value was <0.05 (*p* < 0.05), the differences between groups were significant. IC50 values were evaluated using nonlinear regression in SPSS (Statistical Package for the Social Science, Chicago, Illinois version 22).

## Results

### Preparation of Different Extracts From Barley Bran

#### Percentage Yield of Extracts

After the extraction of 1,010 gm of barley bran, high differences were detected in the percentage yields (Table 3). We used 500 gm of barley bran for ethanol extract, and 200 gm of barley bran for n-hexane, aqueous/methanol, and water extracts. By utilizing the maceration method, the percentage yields for barley branethanol, n-hexane, aqueous/methanol, and water extracts were (1.83%), (0.6%), (2.4%), and (3.5%), respectively. The highest yield was (3.5%) for the water extract, while the lowest yield was (0.6%) for n-hexane extract.

**Table 3:** The percentage yield obtained from the extraction of 1,010 gm of barley bran using the maceration method (% yield = weight after extraction/weight before extraction ^*^ 100%).

**Extraction solvent**	**% of dried extracts yields**
Ethanol	1.83%
*n*-hexane	0.6%
Aqueous/methanol	2.4%
Water	3.5%

#### Qualitative Phytochemical Screening of Barley Bran Extracts

The phytochemical bioactive constituents of ethanol, n-hexane, aqueous/methanol, and water extracts were qualitatively observed using the standard methods (Table 4). The screening results revealed that ethanol extract has phenols and flavonoids. N-hexane extract has terpenoids, steroids, and flavonoids. Aqueous/methanol extract has saponins, phenols, flavonoids, alkaloids, and carbohydrates. Finally, water extract has saponins, tannins, and flavonoids.

**Table 4:** Qualitative phytochemical screening results of secondary metabolites obtained from barley bran extracts.

**Phytochemical screening tests**	**Ethanol extract**	**n-hexane extract**	**Aqueous/** **methanol extract**	**Water extract**
Saponins	–	–	+	+
Tannins	–	–	–	+
Terpenoids	–	+	–	–
Steroids	–	+	–	–
Phenols	+	–	+	–
Flavonoids	+	+	+	+
Alkaloids	–	–	+	–
Carbohydrates	–	–	+	–

*Results were rated as: + (positive), − (negative)*.

#### Quantitative Analysis of Barley Bran n-Hexane and Aqueous/Methanol Extracts by Liquid Chromatography-Mass Spectrometry (LC-MS)

About 5 mg of barley bran n-hexane and aqueous/methanol extracts was utilized for further analysis. This analysis of n-hexane and aqueous/methanol extracts using LC-MS showed the existence of high concentrations of stearic acid, palmitic acid, and 5,6,4'-Trihydroxy-7,3'-dimethoxyflavone with the following percentages: 88.7, 56.37, and 34.3%, respectively (Tables 5, 6). Some other compounds were identified in lower concentration like y-Linolenic acid, 9.3%; 3,5-Dimethoxy-4-hydroxyacetophenone, 4.6%; anthranilic acid, 3.7%; and HexA-Chrysoeriol (or Kaempferide), 3.04%.

**Table 5:** Major compounds identified in barley bran *n*-hexane extract using the LC-MS method.

**No**.	**Compounds**	**Formula**	**RT**	**%**
1	y-Linolenic acid	C18H30O2	29.51	9.316754
2	Stearic acid	C18H36O2	32.82	34.31192
3	Palmitic acid (NMR)	C16H32O2	33.43	56.37133

*RT, retention time*.

**Table 6:** Major compounds identified in barley bran aqueous/methanol extract using the LC-MS method.

**No**.	**Compounds**	**Formula**	**RT**	**%**
1	Anthranilic acid	C7H7NO2	3.14	3.695253
2	3,5-Dimethoxy-4-hydroxyacetophenone	C17H14O7	4.32	4.555799
3	HexA-Chrysoeriol (or Kaempferide) (PUT)	C22H20O12	7.08	3.048765
4	5,6,4'-Trihydroxy-7,3'-dimethoxyflavone	C17H14O7	10.35	88.70018

*RT, retention time*.

### Antiproliferative Activity of Barley Bran Different Extracts on Different Cell Lines

Gradual decreasing in the concentrations of barley bran extracts (5–0.078 mg/ml) on the MCF-7 cell line caused a rise in the average percentage survival in a dose-dependent manner. At concentration of 5 mg/ml, the inhibition percentages of ethanol, n-hexane, and aqueous/methanol extracts were 63, 61, and 57%, respectively. Ethanol, n-hexane, and aqueous/methanol extracts showed the high activity with IC_50_ values of 0.98 mg/ml, 0.77 mg/ml, and 0.85 mg/ml respectively, whereas aqueous extract was the least effective against the MCF-7 cell line with IC_50_ value more than 5 mg/ml (Figures 1A–D).

Similar results were obtained when the HCT-116 cell treated with the same concentrations of barley bran extracts. At concentration of 5 mg/ml, the inhibition percentages of ethanol, n-hexane, aqueous/methanol, and water extracts were 73, 76, 72, and 54%, respectively. Ethanol, n-hexane, and aqueous/methanol extracts showed the high activity with IC_50_ values of.93 mg/ml, 0.38 mg/ml, and 0.67 mg/ml, respectively, whereas aqueous extract was the least effective against the HCT-116 cell line with IC_50_ value of 1.3 mg/ml (Figures 1A–D).

The dose-dependent inhibition was also obtained after treating A549 cells with increasing concentrations of barley bran. At a concentration of 5 mg/ml, the inhibition percentages of ethanol, n-hexane, and aqueous/methanol extracts were 64, 66, and 68%, respectively. Ethanol, n-hexane, and aqueous/methanol showed the moderate activity with IC_50_ values of 2.8 mg/ml, 1.23 mg/ml, and 2 mg/ml, respectively, whereas water extract was the least effective against A549 cell line with IC_50_ value more than 5 mg/ml (Figures 1A–D).

The mouse breast cancer cell line (EMT6/P) was also treated with barley bran, and results showed similar response comparative with other cell lines. At a concentration of 5 mg/ml, the inhibition percentages of ethanol, n-hexane, aqueous/methanol, and water extracts were 61, 69, 71, and 53%, respectively. Ethanol, n-hexane, and aqueous/methanol showed the high activity with IC_50_ values of.84 mg/ml,0.62 mg/ml, and.71 mg/ml, respectively, whereas water extract was the least effective against the A549 cell line with IC_50_ value of 2.2 mg/ml (Figures 1A–D).

The VERO cell line was subjected to various concentrations of extracts, ranging from (5–0.078 mg/ml). The Vero cell line showed more resistance to the tested barley bran signified by the percentage of survived cells. About 66, 64, 60, and 53% survival percentages were reported for ethanol, n-hexane, aqueous/methanol, and water extracts, respectively (Figures 1A–D).

The half maximal inhibitory concentration (IC_50_) is the concentration of a compound causing 50% cell death in comparison to the negative control. IC_50_ values were calculated for all extracts. N-hexane with IC_50_ values of.77 mg/ml, 0.62 mg/ml, 0.38 mg/ml, 1.23 mg/ml was the most effective extract against the MCF-7, EMT6/p, HCT-116, and A549 cell lines, respectively. The toxicity of all extracts was limited with IC_50_ values more than 5 mg/ml. Doxorubicin was utilized as positive control in our antiproliferative trials. Doxorubicin showed good activity against all types of cell lines MCF-7, HCT-116, A549, EMT6/P, and Vero cells lines with respective IC_50_ values of 5.66, 10.84, 101.7, 0.57, and more than 200 μg/ml. Table 7 demonstrates the IC_50_ for all extracts and doxorubicin utilizing five cell lines.

**Table 7:** The IC_50_ of ethanol, *n*-hexane, aqueous/methanol, and water extracts of barley bran using various cell lines in comparison to the normal Vero cell line and to doxorubicin^*^.

**Cell line**	**IC_50_ of ethanol (mg/ml)**	**IC_50_ of *n*-hexane (mg/ml)**	**IC_50_ of aqueous/** **methanol (mg/ml)**	**IC_50_ of water (mg/ml)**	**IC_50_ of doxorubicin (μg /ml)**
MCF-7	0.98 ± 0.22	0.77 ± 0.11	0.85± 0.08	>5	5.66 ± 0.4
EMT6/P	0.84 ± 0.2	0.62 ± 0.4	0.71 ± 0.3	2.2 ± 0.1	0.57 ± 0.4
HCT-116	0.93 ± 0.1	0.38 ± 0.1	0.67 ± 0.1	1.3 ± 0.1	10.84 ± 0.2
A549	2.8 ± 0.1	1.23 ± 0.1	2 ± 0.02	>5	101.7 ± 0.3
VERO	>5	>5	>5	>5	>200

* *Data were presented in (mean ± SD)*.

To identify the safe active concentration for each extract and compare to the VERO normal cell line concentration, selectivity index (SI) also was identified for ethanol, n-hexane, aqueous/methanol, and water extracts of barley bran against MCF-7, HCT-116, A549, and EMT6/P cell lines. SI signifies the ratio between the IC50 of the normal cell line (Vero)/IC50 of each extract. N-hexane extract was mostly effective against HCT-116 and moderately effective against other cell lines. Ethanol and aqueous/methanol extracts were moderately selective against all cancer cell lines except A549. Water extract was the least effective extract against all cancer cell lines. Doxorubicin was the safest against EMT6/P followed by MCF-7 and HCT-116. Table 8 demonstrates the SI of the tested plant extracts.

**Table 8:** The SI of ethanol, n-hexane, aqueous/methanol, and water extracts against MCF-7, EMT6/P, HCT-116, and A549 cell lines.

**Cell line**	**SI of ethanol**	**SI of *n*-hexane**	**SI of aqueous/methanol**	**SI of water**	**SI of doxorubicin**
MCF-7	5.1	6.49	5.88	1	35.3
EMT6/P	5.95	8.06	7.04	2.27	350.8
HCT-116	5.38	13.16	7.46	3.85	18.5
A549	1.79	4.07	2.5	1	2

### Total Phenolic Content (TPC) of Barley Bran Different Extracts Using Folin-Ciocalteu Reagent

The Folin-Ciocalteu procedure was utilized to identify the TPC in barley bran extracts. At 630 nm, the TPC of the extracts was carried out in a concentrations range of 5–0.156 mg/ml.

**Figure 2:**
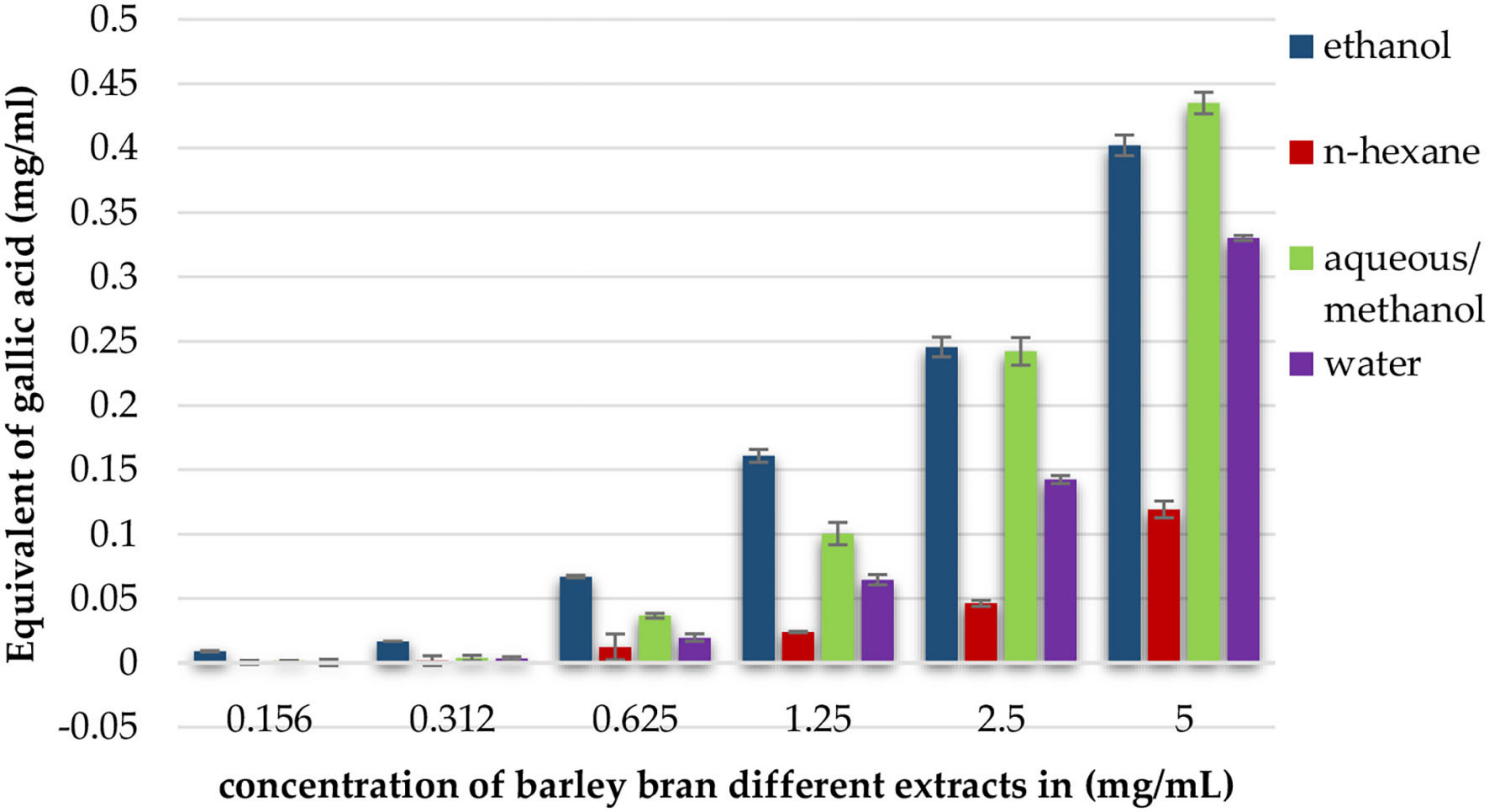
Total phenolic content expressed as equivalent of gallic acid in mg per ml of ethanol, *n*-hexane, aqueous/methanol, and water extracts of barley bran in different concentrations.

At concentrations of 5, 2.5, and 1.25 mg/ml, aqueous/methanol extract showed the highest TPC with 0.44, 0.24, and 0.100 mg/ml equivalent of gallic acid, respectively. Ethanol extract results were 0.40, 0.24, and 0.16 mg/ml at the same mentioned concentrations. The third one in a raw was water extract with values of 0.33, 0.142, and 0.065 mg/ml gallic acid equivalent, and, finally, the lowest content was n-hexane with values of 0.12, 0.046, and 0.024 mg/ml (Figure 2).

### The Effect of Barley Bran Different Extracts on the Proliferation of Splenic Lymphocytes

**Figure 3:**
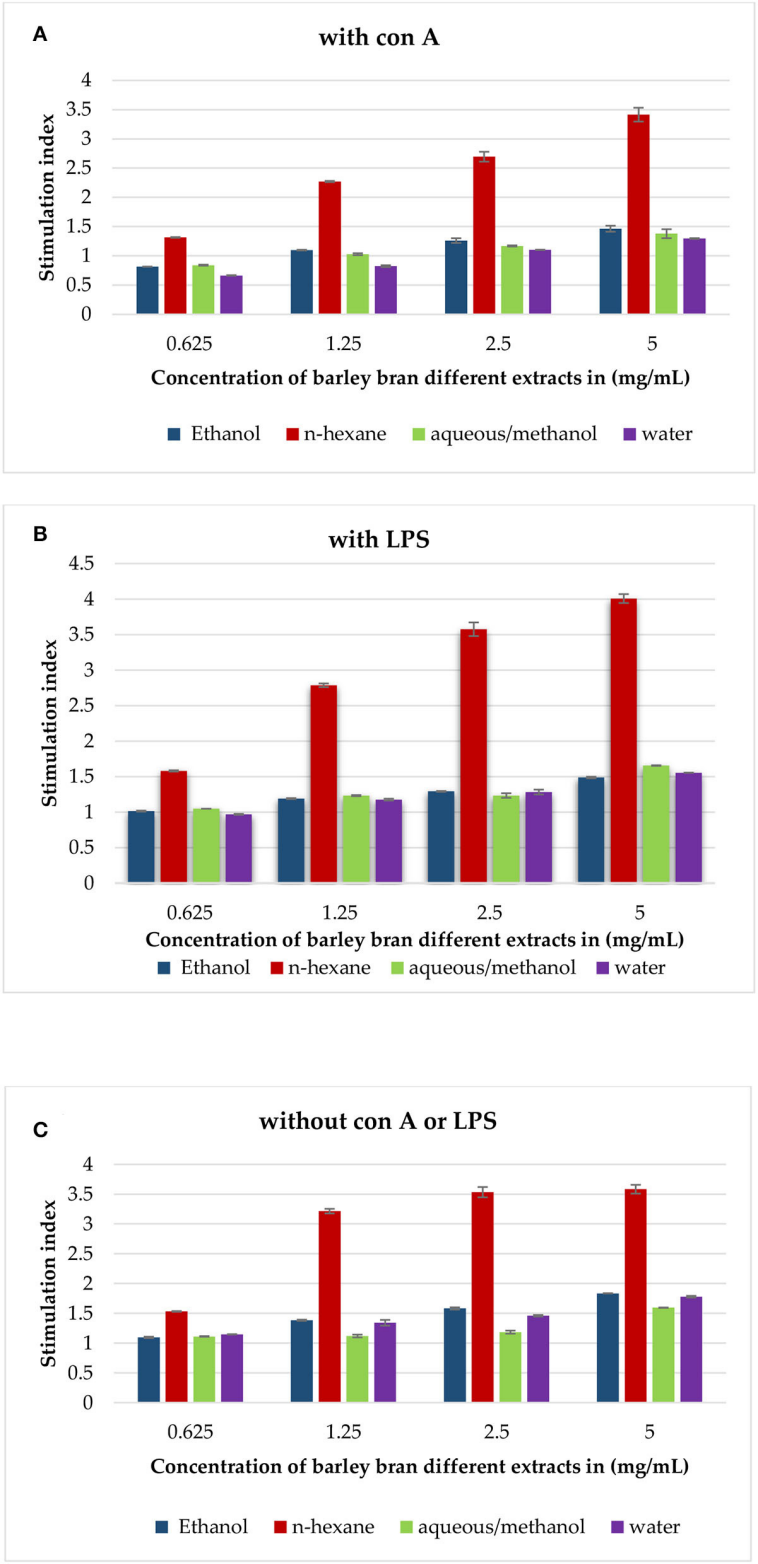
**(A)** The effect of barley bran different extracts at different concentrations on the proliferation of splenic lymphocytes in the presence of Con A (5 μg/ml). **(B)** The effect of barley bran different extracts at different concentrations on the proliferation of splenic lymphocytes in the presence of LPS (4 μg/ml). **(C)** The effect of barley bran different extracts at different concentrations on the proliferation of splenic lymphocytes.

Experimental findings showed that most extracts induced an increase in lymphocytes cell proliferation in the presence and absence of Con A and LPS (Figures 3A–C). In the presence of Con-A and at a concentration of 5 mg/ml, n-hexane extract was the most effective extract, with the highest stimulation index of 3.4, followed by aqueous/methanol and ethanol with stimulation indices of 1.4 and 1.5, respectively.

In the presence of LPS, also n-hexane extract was the most effective extract with the highest stimulation index of 4, followed by aqueous/methanol, aqueous, and ethanol extracts with stimulation indices of 1.7, 1.6, and 1.5, respectively.

The same experiment was repeated without LPS and Con A. Experimental findings showed that all extracts induced an increase in lymphocytes cell proliferation. The stimulation indexes of ethanol, n-hexane, aqueous/methanol, and water extracts were 1.8, 3.6, 1.6, and 1.77, respectively, at concentration of 5 mg/ml (Figure 3C).

### The Effect of Barley Bran Different Extracts on Phagocytosis

**Figure 4:**
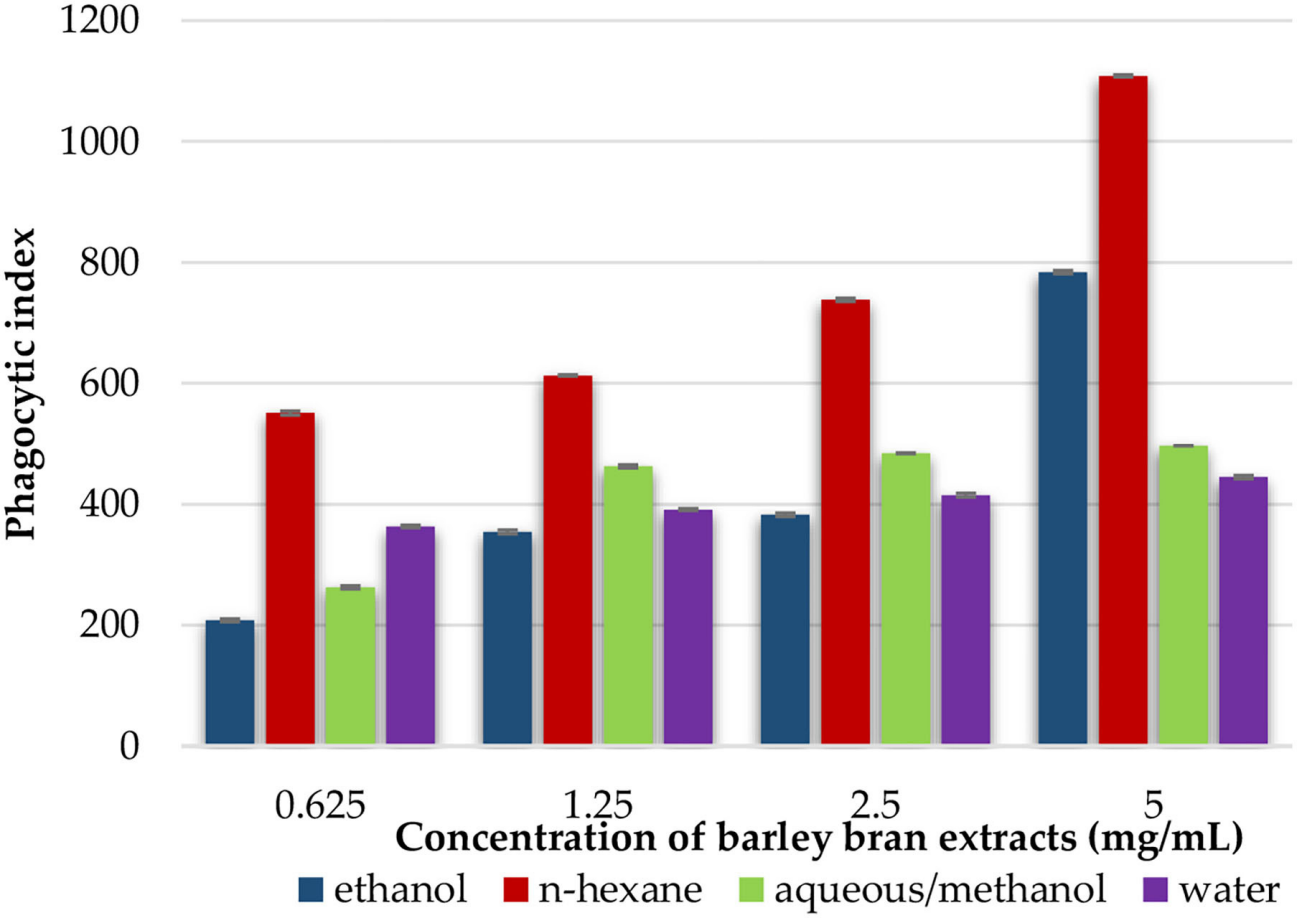
An *in vitro* phagocytic assay using nitro blue tetrazolium (NBT) reduction test of peritoneal macrophage treated with various concentrations of barley bran extracts for 48 h.

Phagocytic activity of peritoneal macrophages was identified by measuring the ability of NBT reduction after treatment with different extracts. We estimated the extracts' effects on phagocytic indices of the cells after 1-h incubation with yeast cells and NBT. The results revealed that peritoneal phagocytic activity was significantly increased after exposure to the extracts at concentration that ranged from 5 to 0.625 mg/ml. At concentration of 5 mg/ml, n-hexane had the highest increase with 1,108% of phagocytic activity, followed by ethanol with 784% and aqueous/methanol with 497%, while water extract had a lower activity at the same concentration with phagocytic index of 445% (Figure 4).

### The Effect of Barley Bran Different Extracts on Pinocytosis

**Figure 5:**
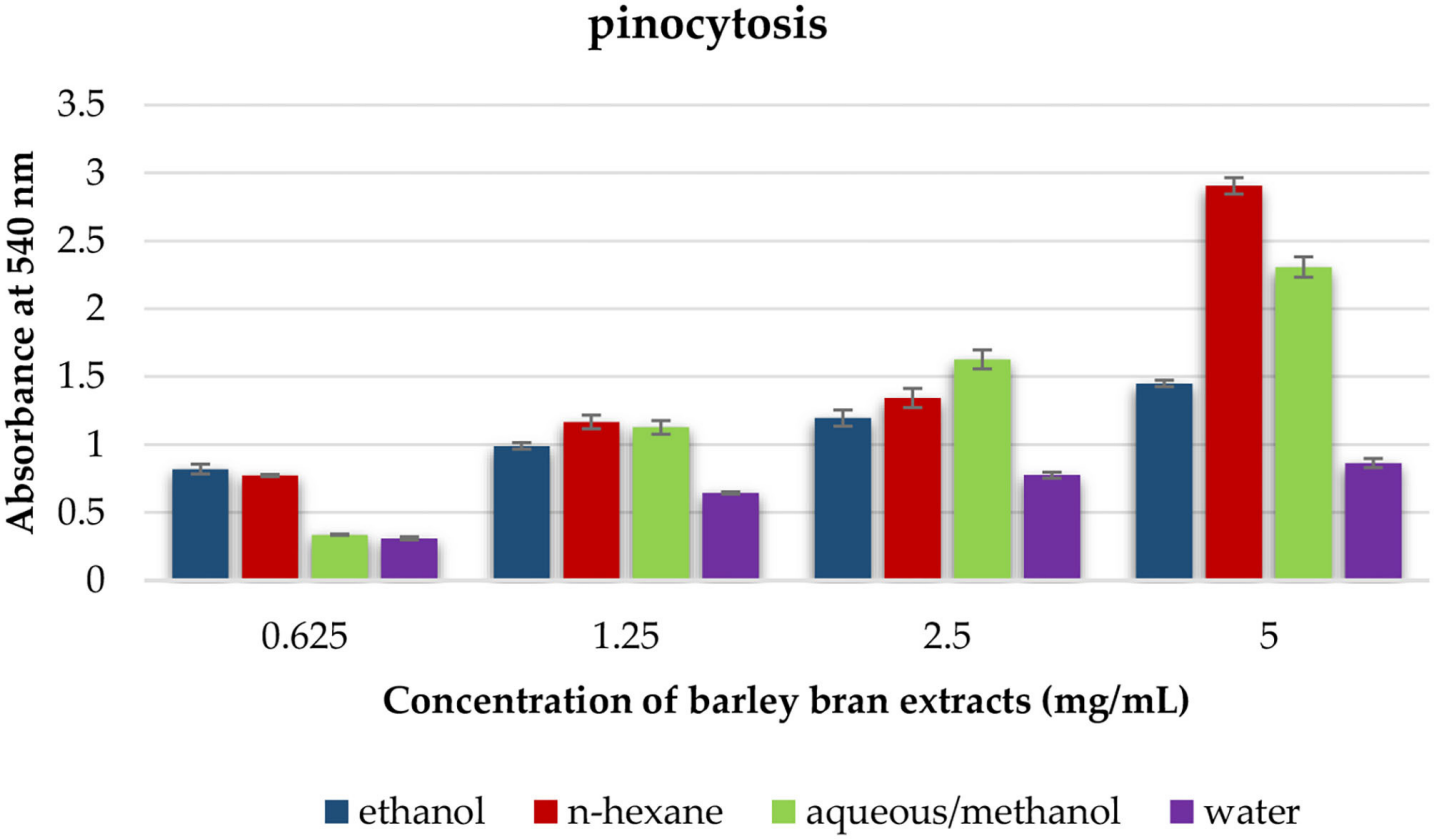
The effect of barley bran different extracts on macrophage pinocytosis.

This test was utilized to estimate the effect of each solvent extract on the pinocytotic activity of macrophages. At a concentration that ranged within 5–0.625 mg/ml, the pinocytotic activity was enhanced under exposure to different extracts. N-hexane extract produced the highest increase in the pinocytotic activity with value of 2.9 compared with control value of 0.8. However, the pinocytotic activity of aqueous/methanol, ethanol, and water extracts was 2.3, 1.5, and 0.9, respectively (Figure 5).

### Part Two: *In vivo* Results

#### Effect of Barley Bran Consumption on Tumor Development and Growth

After Balb/C mice were fed fodders having various percentages of barley bran for 15 days, tumor inoculation was performed with the EMT6/p cell line subcutaneously. After that, we waited for 15 days with the continuing of feeding until the day of the sacrifice. Caliper digital was utilized to measure the tumor sizes, and then the tumors were weighed at the end of the study.

**Figure 6:**
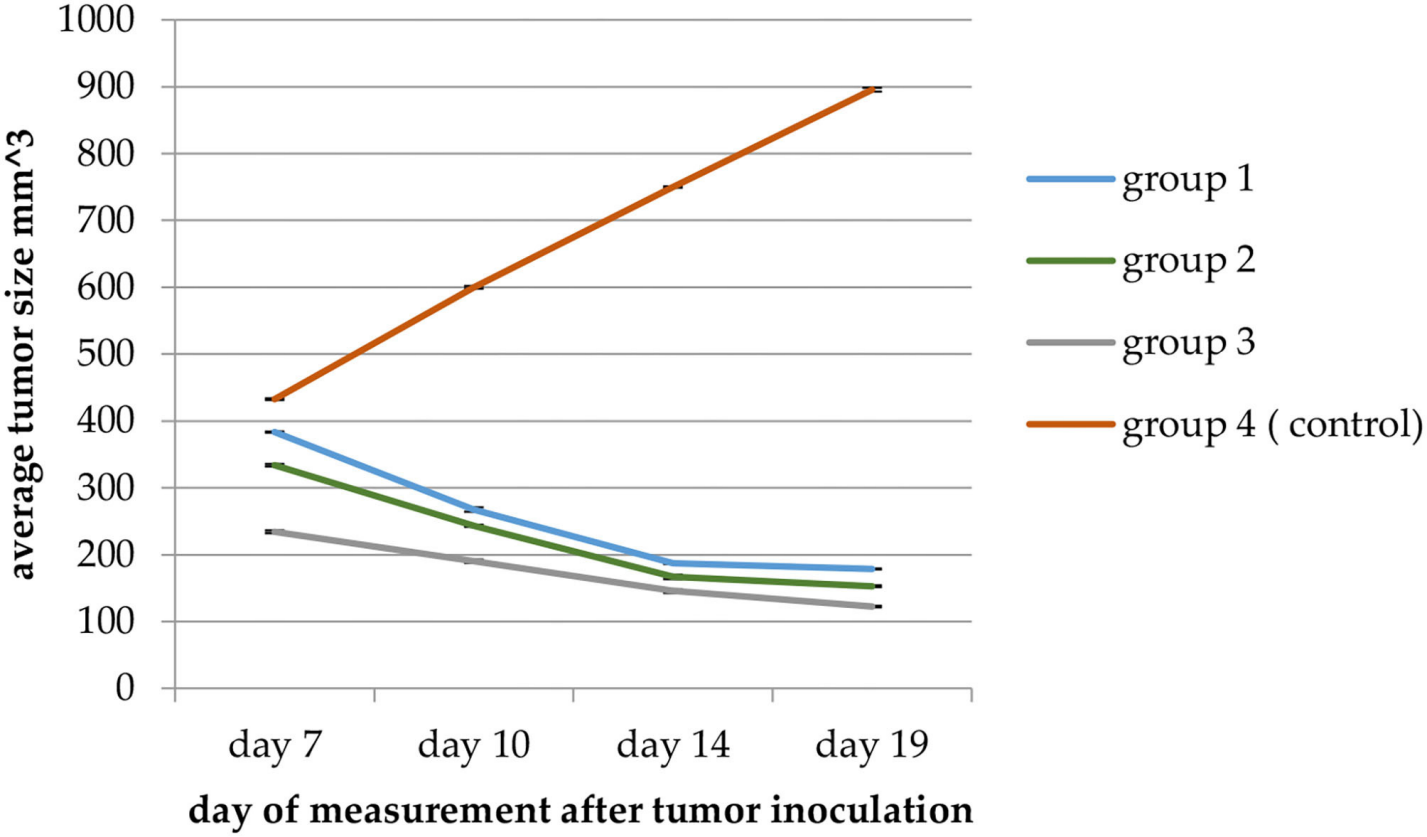
A plot of change in average tumor size (mm3) vs. time in (days) of measurement in the EMT6/P cell line. Average tumor size in Group 3 (mice given fodders with 30% barley bran) was significantly (*p* < 0.05) reduced compared with the control group.

**Figure 7:**
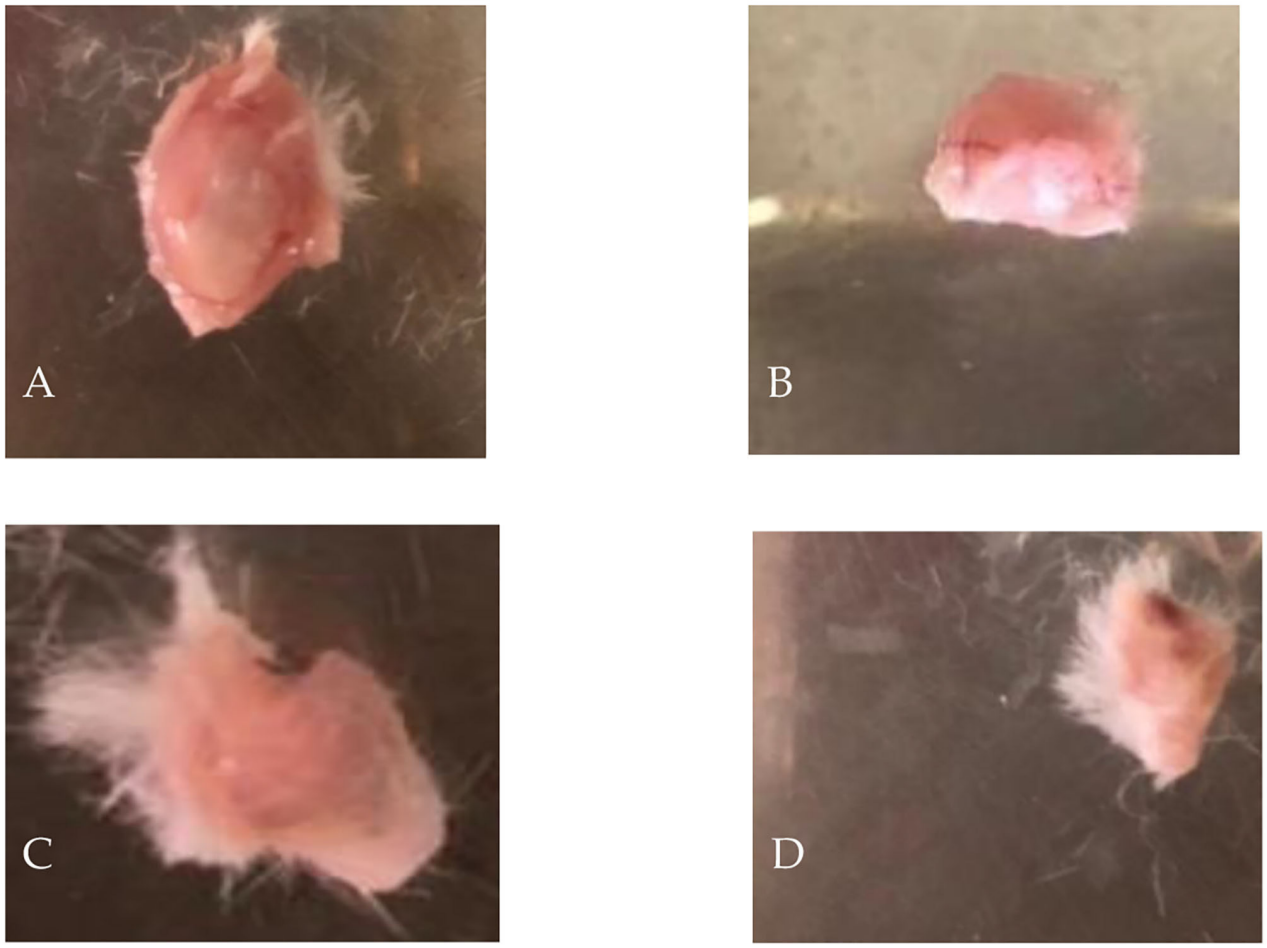
Dissected EMT6/P tumors showing: **(A)** negative control, **(B)** given 10% of barley bran, **(C)** given 20% of barley bran, **(D)** given 30% of barley bran.

Tumor size in mice given fodders with barley bran was significantly (*p* < 0.05) reduced compared to the control group (Table 9). Feeding 10, 20, and 30% barley bran with fodders showed significant inhibition of tumors where a reduction in tumor size was reported −53.4, −54.2, and −47.7%, respectively. These values are significantly lower than the negative control group, which showed an increase in tumor size by 107.02% (Figure 6). Only one case of death was recorded for the control group. Groups-treated barley bran showed no mortality. The percentages of mice with no detectable tumor in groups Numbers 1 (10% BB), 2 (20% BB), and 3 (30% BB) were 20, 20, and 30%, respectively. However, the percentage of the mice that showed no tumors after tumor inoculation was 30%. The mice exhibited normal activity without side effects (Figure 7).

**Table 9:** The effect of barley bran on tumor size and weight in mice (*n* = 10) (mm3: cubic millimeter).

**Group**	**Av. initial tumor size (mm3) ±SEM**	**Av. final tumor size (mm3) ±SEM**	**% change in tumor size**	**% of mice with no detectable tumor**	**Av. tumor weight (g)**
1: 10% BB; *n =* 10	383.4056 ± 2.47	178.646 ± 0.102	−53.4055	20%	0.190
2: 20% BB; *n =* 10	333.83345 ± 2	152.6542 ± 1.6	−54.2724	20%	0.172
3: 30% BB; *n =* 10	234.1909 ± 2.6	122.4254 ± 2	−47.7241	30%	0.082
4: Control; *n =* 10	432.62 ± 1.19	895.61 ± 1.08	107.02	10%	0.72

#### The Effect of Barley Bran on the Serum Levels of IFN-γ, IL-2, IL-4, and IL-10

**Figure 8:**
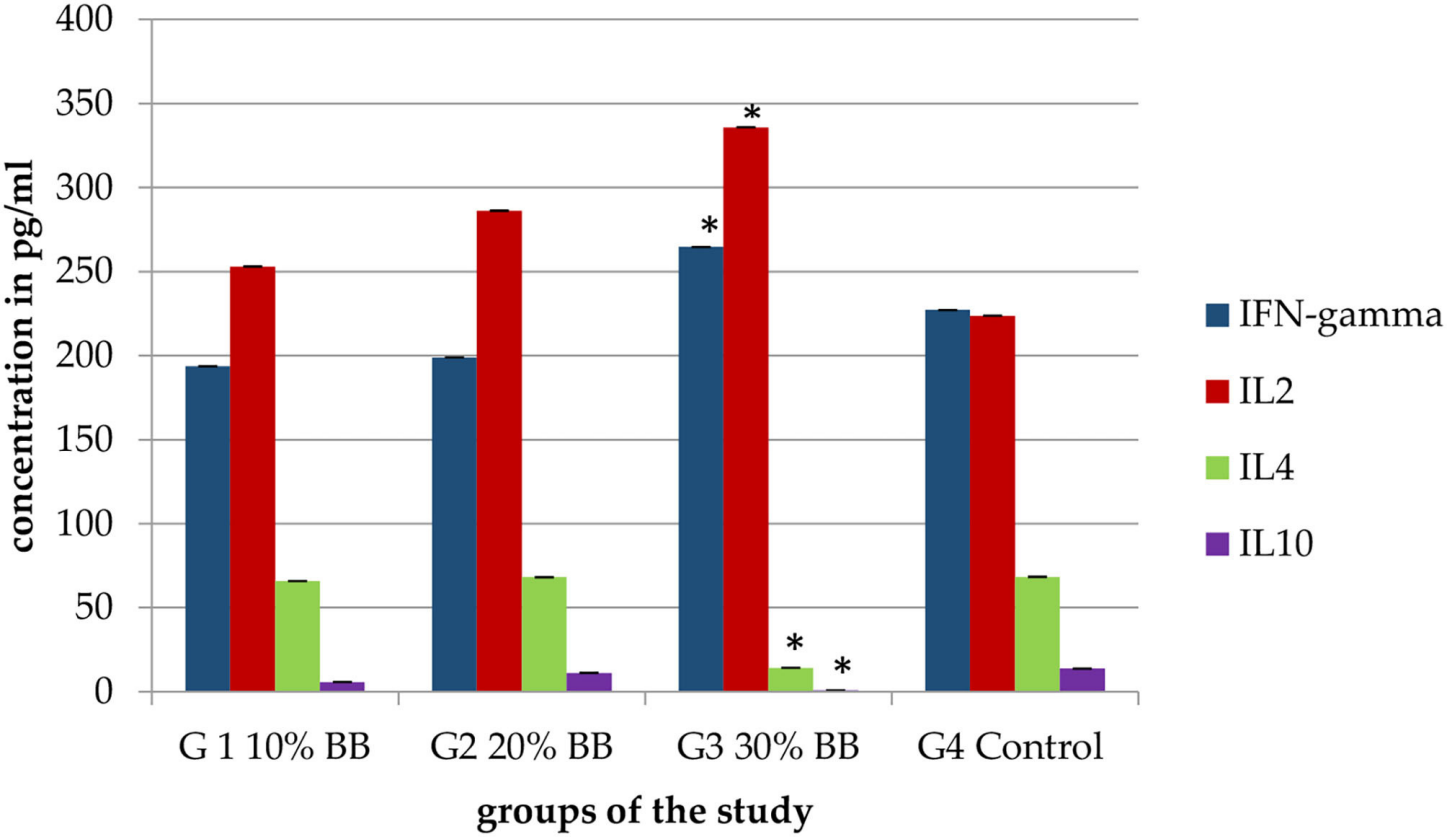
Serum levels of IFN- γ, IL-2, IL-4, and IL-10 for the mice treated with increasing levels of barley bran. The G3 30% barley bran group significantly (*p* < 0.05) induced higher levels of IFN- γ and IL-2 and lower levels of IL-4 and IL-10 compared with the control group. The * symbol indicates the significant values.

Three mice of each group were utilized to take serum samples to measure the level of cytokines and compare them with the control group. The use of barley bran resulted in change in the cytokine levels. IFN-γ, IL-2, IL-4, and IL-10 values of 30% barley bran group were 265 0, 336 0, 14 0, and 0.8 pg/ml, respectively. While IFN-γ, IL-2, IL-4, and IL-10 values of the control group were 227, 224, 68, and 14 pg/ml, respectively (Figure 8), meaning that the 30% barley bran group induced higher levels of IFN- γ and IL-2 and lower levels of IL-4 and IL-10 compared with the control group.

## Discussion

Increased emphasis has now been put on complementarity and alternative medicine (CAM) anti-cancer therapies due to the rapid rise in cancer incidence and mortality. Medicinal herbs have been used in conjunction with chemotherapy and radiotherapy as adjunctive therapy to minimize drug resistance and side effects such as osteoradionecrosis (14). Barley bran has secondary metabolites, such as saponins, tannins, phenols, flavonoids, alkaloids, terpenoids, steroids, and carbohydrates that play a crucial role in cancer prevention (14). In this study, crude extracts of barley bran were prepared and assessed for their anticancer and immunomodulatory effect. Ethanol, n-hexane, aqueous/methanol, and water extracts were produced *in vitro* by maceration and examined for their antiproliferative activity against MCF-7, HCT-116, A549, EMT6/p, and the VERO normal monkey kidney cell line. On the other hand, an *in vivo* study was performed to examine the prophylactic and antitumor effects of the barley bran on female Balb/C mice inoculated with the EMT6/p breast cancer cell line. Barley bran extracts were capable to inhibit tumor progression *in vitro* and *in vivo* experiments.

In a dose-dependent way, barley bran extracts showed potent inhibitory effects on cancer cells. Based on the registered results, n-hexane and aqueous/methanol extracts inhibited the cellular growth of all tested cancer cell lines (Figures 1B,C). Ethanol extract was active against all cancer cell lines; however, water extract was only active against EMT6/p and HCT-116 cell lines (Figures 1A,D).

As quantitative phytochemical screening tests were performed for all extracts, the phytochemical active constituents were revealed. N-hexane extract has terpenoids, steroids, flavonoids, and carbohydrates, while aqueous/methanol extract has saponins, phenols, flavonoids, alkaloids, and carbohydrates. Further analysis was applied using liquid chromatography-mass spectrometry (LC-MS) to determine the most important phytochemicals in n-hexane and aqueous/methanol extracts (Tables 5, 6). These findings are consistent with previously conducted study, which revealed phenolic acids, flavonoids, lignans, tocols, and phytosterols as phytochemicals in barley (14). These phytochemicals affect cancer through the regulation of various molecular pathways involved in tumor growth and progression. Proliferation inhibition, cell cycle arrest, apoptosis induction, immune system modulation, and improvement of the antioxidant environment are the particular mechanisms of phytochemicals (26). By modulating the levels of reactive oxygen species (ROS) in cells, herbal extracts rich in phenolic compounds can control cell proliferation, survival, and apoptosis. Also, plant phenolic acids played an important role in inhibiting cancer growth in several *in vitro* observations, preclinical and epidemiological studies (27). Our qualitative phytochemical screening revealed the presence of phenolic compounds in many extracts. Reactive oxygen species (ROS) regulate cell proliferation, survival, and apoptosis by causing genetic mutations, increasing oxidative stress, and activating oncogenes (28). A previous study showed that barley seeds extracts have antioxidant activities (29), which might explain the ability of our extracts to inhibit the viability of cancer cells.

Guneidy et al. (30) showed that there were large preventive effects and small cytotoxic effects of flavonoids on various cancer cell lines, for instance, the MCF-7 cell line. Flavonoids were also effective against A549, and HCT-116. Li et al. (31) revealed that the flavonoids can reduce cell proliferation and metastasis; also, they can strengthen apoptosis. Moreover, trihydroxy-methoxyflavone showed antiproliferative activity against human leukemia cells through inducing apoptosis (cell-programmed death), and activating the MAPK pathway (32). As the four extracts contained flavonoids, the previous study confirmed the ability of all extracts to have antitumor properties.

Thoppil and Bishayee (33) showed the cytotoxic and chemopreventive effects of terpenoids on different cancer cell lines. Also, Bardon et al. (34) showed the inhibition effect of terpenoids on tumor growth. In order to demonstrate the antiproliferative activity of n-hexane against all tested cancer cell lines, Kumar et al. (35) showed that n-hexane was utilized as a main solvent in the terpenes extraction. Moreover, alkaloids are essential phytochemicals in plants. Several alkaloids isolated from natural herbs possessed anti-metastasis and anti-proliferation effects on various cancer forms, both *in vitro* and *in vivo* (36). Also, Talib (37) showed that the cellular growth of EMT6/p was inhibited by alkaloids. Our results showed that flavonoids and carbohydrates were found in both n-hexane and aqueous/methanol extracts, which confirms the potent cytotoxic effects of these two extracts against different cancer cell lines.

Barley is regarded as a good phytosterol source, even though the phytosterol level of barley is moderate compared to other major grains (38). Awad and Fink (39) showed that natural dietary plant sterol intake can have a beneficial impact and may prevent cancer of the colon, prostate, and breast. Sitosterol is the most abundant type of sterol in barley, as in most grains (38). Numerous mechanisms of action against cancer were proposed for phytosterols, including activation of apoptosis, reduction of carcinogen production, and stimulation of the sphingomyelin cell cycle (40). Beta glucans are complex carbohydrates made up of polysaccharides. Beta-glucans were utilized to slow cancer growth and prevent it from spreading to other parts of the body (41). In our study, n-hexane and aqueous/methanol extracts possessed potent cytotoxic activity, which can be explained by the presence of steroids and carbohydrates, respectively.

In our study, saponins were detected in aqueous/methanol extract. Geronimo et al. (42) showed that the saponins inhibited the cellular growth of different cancer cell lines. Saponins and tannins possessed antitumor properties in MCF-7 and HCT-116 (42).

As we made Folin-Ciocalteu procedure, total phenolic content was highest in aqueous/methanol. The main phenolic groups in barley are ferulic acid, vanillic acid, syringic acid, and p-coumaric acid (14). A previous study showed that barley can act as an excellent natural diet for its anti-proliferative capacity because it has abundant content of phenolic acids (14). Anthranilic acid derivatives inhibited different cancer cell lines, including A549. A study revealed that γ-Linolenic acid exerted potent inhibitory effect on pheochromocytoma cancer cells (43). Palmitic acid was able to reduce cell viability in MCF-7 breast cancer cells by enhancing the expression of apoptosis-related proteins, including caspase-3, 9, Bax, and P53 (44). These results confirm the cytotoxicity effect of aqueous/methanol and n-hexane extracts, respectively.

Vero cells are cell lineages that were first isolated from kidney epithelial cells taken from an African green monkey (Cercopithecus aethiops). Yasumura and Kawakita developed the lineage in 1962. The original cell line was termed “Vero” after an acronym of verda reno, which means “green kidney”(45). The Vero cell lineage is continuous and aneuploid, which means it contains an aberrant number of chromosomes. A continuous cell lineage can be replicated through many cycles of division without becoming senescent. Vero cells have the interferon-alpha/beta receptor; thus, they respond appropriately when recombinant interferon is given to their culture media (45). Ethanol, n-hexane, aqueous/methanol, and water extracts of barley bran showed limited toxicity against the VERO cell line with IC_50_ value more than 5 mg/ml. This may demonstrate the safety of all extracts against normal cells.

The immune system is a complex network of cells and proteins that defend the body against invading microorganisms and against tumor cells. Immune system modulation can be achieved through a range of specific and non-specific approaches. Immunomodulation refers to any changes in the immune system, and it can be induction, expression, amplification, or inhibition of any part in the immune system (46). Lymphocytes are a critical part of the acquired immune system, and their ability to proliferate is seen as a measure of the extent of cell immunity (46).

According to our findings, the proliferation of both T and B lymphocytes was increased by barley bran extracts with some variation. The rising level of splenic lymphocytes upon using barley bran indicates an immune-stimulatory effect on the acquired immune system.

Either in the presence of Con A or LPS, n-hexane extract was the most effective extract with the highest stimulation index followed by aqueous/methanol (Figures 3A,B). Also, n-hexane extract was the most effective extract in the absence of both Con A and LPS. These findings may be clarified by the fact that most of the phytochemicals existed in plants had immune-stimulating activity (47). It was reported that methoxylated flavonoids have the ability to inhibit CYP1B1 activity and mRNA expression in human oral squamous cell carcinoma SCC-9 cells (48). Terpenoids had anticancer and immune-stimulating activities (49). A study showed that the percentage of T and B cells along with the induction of Splenocyte proliferation in both resting and LPS-stimulated cells was risen by plant-derived polyphenols (50). Saponins were utilized to increase lymphocyte proliferation, enhance cytolytic activity of natural killer cells (NK), and produce a high CD4+/CD8+ ratio (51). Furthermore, the immunostimulatory effects of carbohydrates derived from barley leaf were revealed by enhancing splenocytes proliferation and NK cytotoxic activity with a significant rise in Th1 cytokines (52). Stearic acid derivatives can influence the anti-inflammatory activity through the inhibition of NO and TNF-α. Palmitic acid, a saturated fatty acid, significantly upregulates the expression of signaling lymphocytes-activation molecule family Member 3 through the JAK/STAT5 pathway as well as increased the induction of TNF-α, IL-1β, IL-6, IFN-γ, IL17A, and IL-2 (53). γ-Linolenic acid has been proved to boost lymphocytes, which are important components of immunological response. γ-Linolenic acid may also have crucial roles in cancer treatment. It demonstrated some improvement in immunologic status in patients with estrogen-sensitive breast cancer and bladder cancer (54). The relevance of γ-Linolenic acid stems from the fact that it is the precursor of prostaglandins, leukotrienes, and thromboxanes, which operate as mediators of inflammation and immunological processes in diseases like cancer, diabetes, arthritis, cardiovascular disease, and cellular aging (55). Other study showed that tricin, a type of trihydroxy-methoxyflavone, possessed antiangiogenic activity *in vitro*. It was able to reduce VEGF expression, inhibit HIF-1α accumulation in tumor cells, and modulate ROS generation in endothelial cells (52).

The effects of barley bran on innate immune system were evaluated by utilizing phagocytic activity test and pinocytic activity test. Endocytosis includes both phagocytosis (cell eating) and pinocytosis (cell drinking). The frontline defensive mechanism against pathogen invasion and a crucial part of the innate immune system was phagocytosis. Macrophages were recognized for their phagocytic activity and capacity to polarize into phenotypes, which are pro-inflammatory (M1) and anti-inflammatory (M2) (56). During carcinogenesis, M1-like polarization of anti-tumor macrophages was involved in the removal of more immunogenic tumor cells (57). This study showed that barley bran has the ability to increase the phagocytic activity of macrophages in a dose-dependent manner. N-hexane extract was the most active, followed by ethanol extract, while aqueous/methanol and water were less active (Figure 5).

The high immune-stimulating effect of n-hexane extract might be due to the high content of flavonoids. A previous study showed that oral flavonoids were utilized to enhance the activity of macrophages through converting β-glucuronidase to aglycones in macrophages (58). Immunostimulation in γ-Linolenic acid was characterized by a rise in phagocyte oxidative burst, CD4 + CD8-lymphocytes in blood, and the CD4: CD8 ratio (59). Stearic acid can stimulate the NF-κB pathway via TLR4 signaling (60). Other studies have shown that palmitic acid was able to enhance activation of TLR4 and NF-?B pathways as well as increase secretion of IL-18, TNF-α, IL-1β, and activation of TLR2 (61). Immunostimulation activity and phagocytic induction were revealed by phytosterols by increasing ROS and enhancing the production of NO (62).

For pinocytic activity assay, our findings revealed that the activity of pinocytosis of barley bran was increased in a dose-dependent model. N-hexane and aqueous/methanol showed high activity compared to control (Figure 5).

An *in vitro* study revealed that plant-derived polysaccharides could stimulate murine lymphocyte proliferation, increase murine pinocytic activity, and enhance releasing of nitric oxide (NO), IL-1β, and TNF-α in macrophages (63). Palmitic and stearic acids can activate inflammatory pathways in microglia, as evidenced by an increase in pro-inflammatory cytokine production (IL-1β and TNF-α) (60). Other study demonstrated that the phytosterols stimulated phagocytosis and pinocytosis activity (64). These facts may describe the pinocytic activity of n-hexane and aqueous/methanol extracts of barley bran.

For the *in-vivo* part, the size of breast tumor was reduced compared to the control group with the usage of 30% bran fodder. This reflects the prevention ability of barley bran usage against breast cancer. Reddy et al. (65) revealed the prevention effect of bran on colon cancer in rats when bran oil was utilized with 2% in their food, leading to a significant decrease in the incidence of cancer when it was fed before and after inoculation. Other study showed that the rats were fed 20% wheat bran fodder and a significant decrease in the size of the colon cancer foci was revealed (66). Using tramp mice as a prostate cancer model, Carter et al. (67) found that the incidence of cancer was decreased significantly due to its antioxidant effect by feeding them wheat bran diet in different concentrations.

Xiao et al. (68) listed the prevention of breast cancer and how the estrogen binding capacity of wheat bran could contribute to prevent breast cancer.

In determining the influence on cytokine's level, the 30% group showed higher concentration of IFN-gamma and IL-2 compared with the negative control group. IFN-gamma and IL-2 are Th1 cytokines, while IL-4 and IL-10 are Th2 cytokines. A balanced ratio of Th1/Th2 cytokines is observed in healthy humans. Increased concentrations of Th2 cytokines were observed in patients with different tumor types. Inhibition of Th2 cytokines and enhancement of Th1 were significantly observed in the 30% barley bran group. It revealed the influence of arabinoxylans present in the bran. Arabinoxylans had an immune boosting effect when Balb/c mice were fed enzymatically modified bran. Li et al. (69) showed that ergosterol has the ability to potentiate the immune system. Kumar et al. (70) revealed the immunomodulatory activity of terpenoids. The immunomodulatory and antitumor activities of carbohydrates were demonstrated by Gajos et al. (71). A previous study showed that the flavonoids could activate the secretion of IFN-γ and IL-2 (72). Another study revealed that the production of interferon (IFN)-g was induced by beta-glucan.

Barley contains about 17% fiber, which is one of the highest percentages of any whole cereal grain. Fiber in barley bran includes beta glucan, arabinoxylan, and lignin. Barley bran fiber has been proved to improve glycemic response, blood lipid attenuation, intestinal enzymatic activity, dietary digestibility, and gut flora (73).

Fiber can aid in the prevention of constipation and diarrhea by generating a bulk within the digestive tract and so regulating bowel movements. A study investigated the impact of adding more barley to adult women's diets and discovered that, after 4 weeks, barley intake had a positive influence on both lipid metabolism and gastrointestinal function (74). Fiber is also necessary for a healthy bacterial balance in the digestive tract. It essentially feeds probiotic bacteria in the gut, assisting in the production of short-chain fatty acids, such as butyrate, which have anti-inflammatory properties and may aid in the treatment of symptoms associated with irritable bowel syndrome, Crohn's disease, and ulcerative colitis (74).

Lignan may offer protection against the development of cancer and heart disease by assisting the body in metabolizing bacteria and maintaining a healthy ratio of “good-to-bad” bacteria within the gut, hence reducing overall inflammation (73). Lignans, polyunsaturated fatty acids, oligosaccharides, plant sterols, and saponins are components found in barley bran that can help fight free radical damage and inflammation. These compounds have mechanistic actions that include binding to and eliminating toxic carcinogens from the body (73).

The synergistic action of barley bran components can aid in the anticancer and immunomodulatory properties of barley bran.

## Conclusion

The present study was performed to evaluate the anticancer and immunomodulatory activities of barley bran. The outcomes revealed that the barley bran can diminish cancer cell viability and boost the immune system. The findings of this study revealed the immunomodulatory activity of barley bran by prompting splenic lymphocytes proliferation, phagocytosis, and pinocytosis. Also, when we tested the bran *in vivo*, the prevention percentage and the small-scale-produced tumors were a good simulation for the prevention effect of barley bran in reality. The presence of various phytochemicals in barley bran can explain its stimulator effect for the innate and acquired immune system.

## Data Availability Statement

The original contributions presented in the study are included in the article/Supplementary Material, further inquiries can be directed to the corresponding author/s.

## Ethics Statement

The animal study was reviewed and approved by Research and Ethical Committee at the Faculty of Pharmacy-Applied Science Private University.

## Author Contributions

All authors listed have made a substantial, direct, and intellectual contribution to the work and approved it for publication.

## Funding

The authors are grateful to the Applied Science Private University, Amman, Jordan for the full financial support granted to this research.

## Conflict of Interest

The authors declare that the research was conducted in the absence of any commercial or financial relationships that could be construed as a potential conflict of interest.

## Publisher's Note

All claims expressed in this article are solely those of the authors and do not necessarily represent those of their affiliated organizations, or those of the publisher, the editors and the reviewers. Any product that may be evaluated in this article, or claim that may be made by its manufacturer, is not guaranteed or endorsed by the publisher.
