# Supplementary material for: Immunomodulatory and Anticancer Activities of Barley Bran Grown in Jordan: An in vitro and in vivo Study
Source: Front Nutr. 2022 May 18;9:838373. doi: 10.3389/fnut.2022.838373 (PMC9159360; doi:10.3389/fnut.2022.838373)
Supplement: Supplementary file 1 [file Data_Sheet_1.docx]

**Appendices**


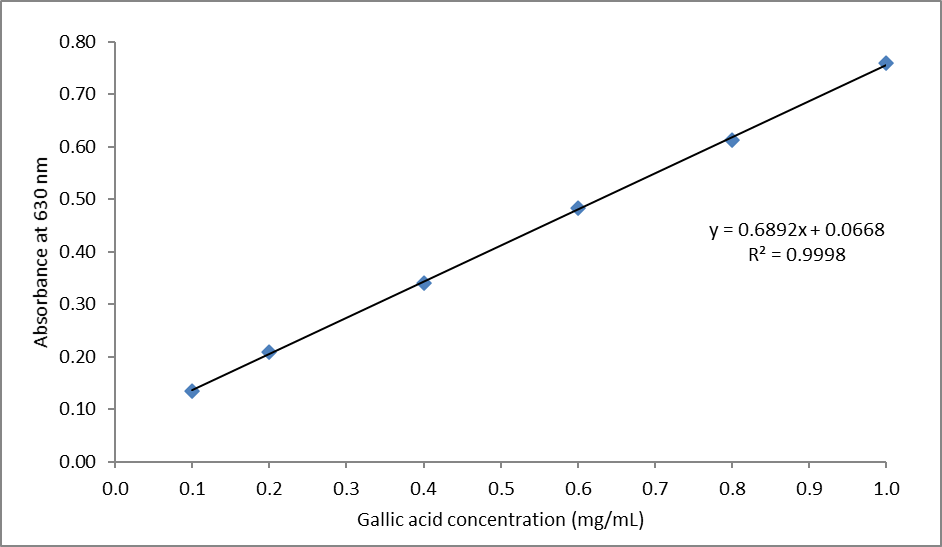


**Appendix (3.1): standard curve for gallic acid.**


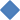

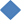

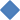

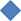

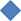

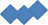


1.3

1.2

1.1

1

0.9

0.8

0.7

0.6

0.5

0.4

0.3

0.2

0.1

0

y = 0.0022x + 0.1059

R² = 0.9961

0 50 100 150 200 250 300 350 400 450 500 550

Mouse IFN-γ Concentration (pg/ml)

OD=450nm

**Appendix (3.2): standard curve for mouse IFN-γ**


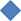

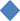

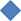

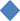

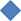

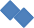


1.8

1.7

1.6

1.5

1.4

1.3

1.2

1.1

1

0.9

0.8

0.7

0.6

0.5

0.4

0.3

0.2

0.1

0

y = 0.0028x - 0.0184

R² = 0.9923

0 50 100 150 200 250 300 350 400 450 500 550 600 650

Mouse IL-2 Concentration (pg/ml)

OD=450nm

**Appendix (3.3): standard curve for mouse IL-2.**


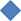

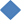

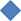

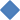

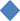

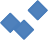


1.4

1.3

1.2

1.1

1

0.9

0.8

0.7

0.6

0.5

0.4

0.3

0.2

0.1

0

y = 0.0022x + 0.1651

R² = 0.9955

0 50 100 150 200 250 300 350 400 450 500 550

Mouse IL-4 Concentration (pg/ml)

OD=450nm

**Appendix (3.4): standard curve for mouse IL-4.**


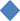

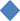

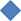

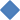

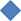

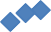


1.5

1.4

1.3

1.2

1.1

1

0.9

0.8

0.7

0.6

0.5

0.4

0.3

0.2

0.1

0

y = 0.0024x + 0.178

R² = 0.9961

0 50 100 150 200 250 300 350 400 450 500 550

Mouse IL-10 Concentration (pg/ml)

OD=450 nm

**Appendix (3.5): standard curve for mouse IL-10.**
